# Comparison of Different Solid-Phase Cleanup Methods Prior to the Detection of Ciguatoxins in Fish by Cell-Based Assay and LC-MS/MS

**DOI:** 10.1021/acs.jafc.5c01142

**Published:** 2025-06-02

**Authors:** Andres Sanchez-Henao, Fernando Real, Yefermin Darias-Dágfeel, Natalia García-Álvarez, Jorge Diogène, Maria Rambla-Alegre

**Affiliations:** † 16750 IRTA, Marine and Continental Waters Program, Carretera de Poble Nou, 43540 La Ràpita, Catalonia, Spain; ‡ IUSA, University Institute of Animal Health and Food Safety, University of Las Palmas de Gran Canaria, C/Trasmontaña S/N, Arucas, Canarias 35413, Spain

**Keywords:** ciguatoxins, cell-based assay, LC-MS/MS, solid phase extraction, cleanup, fish

## Abstract

Ciguatera poisoning (CP) is the most reported food poisoning associated with fish consumption. Ciguatoxins (CTXs) are produced by microalgae and metabolized in fish; even low levels of these toxins in fish can lead to CP. To date, there is no unique validated methodology for their study, and demonstrating their presence in fish tissues is an analytical challenge. The main techniques used are cell-based assay and liquid chromatography, which may present different matrix effect interferences; thus, purification protocols are necessary. Six cleanup strategies for fish extracts, assessing the principal analogues found in fish in different parts of the world (CTX1B/CTX3C/C-CTX1), are compared here. Cleaned-up extracts are evaluated by cell-based assay and chromatography. All protocols are suitable for recovering the analogues of CTXs. Two of them, those that used polystyrene-divinylbenzene and silica cartridges, achieve the most adequate results showing toxicity in their fractions over 53% and chromatography efficiencies over 79% for CTX1B/CTX3C, proving to be the most versatile clean-ups for the study of the different CTX analogues.

## Graphical abstract

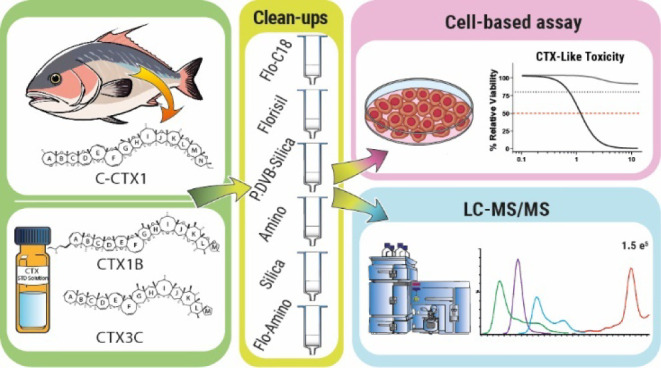

## 1 Introduction

Ciguatoxins (CTXs) are a group of polyether compounds naturally produced by dinoflagellates from the genera Gambierdiscus and Fukuyoa, present mainly in tropical and subtropical waters. These toxins are incorporated in the trophic web by herbivorous and omnivorous fish, and also invertebrates, which are then preyed upon by carnivorous fish. Through trophic webs, the toxins are accumulated and biotransformed, resulting in different CTXs, potentially more toxic. The consumption of these contaminated animals leads to the human poisoning known as ciguatera.^1^ To date, more than 30 analogues of CTXs have been described,^1–4^ and they are traditionally classified according to the geographic area where they were produced: Pacific CTXs, Caribbean CTXs, and Indian CTXs.^5^ However, this nomenclature tends to be replaced by a classification based on their chemical structure, since there is no clear regional limit for their appearance.^1,6,7^ Ciguatoxins are of great importance in the scientific, health, and socioeconomic fields due to their toxic potential. European legislation establishes that fishery products containing CTXs must not be placed in the market, without setting a maximum permitted level.^8^ There is no certified and validated method for CTX analysis,^9^ leaving an action gap that the laboratories responsible for preventing these products from reaching consumers have to face, as is the case in the Canary Islands.^10,11^ Therefore, it is necessary to develop methodologies that ensure the detection of low levels of CTXs in a way that guarantees food safety. In this regard, the European Food Safety Authority (EFSA) and the Food and Drug Administration (FDA) include in their texts a guidance limit of 0.01 pg equivalents (Eq) of CTX1B/g of fish tissue for safe consumption.^12,13^ Furthermore, new reports suggest that this limit is insufficient.^1^ Nonetheless, not all the available methodologies are capable of achieving this level of sensitivity. On the one hand, cell-based assay (CBA) is a useful tool for toxicity evaluation; however, this method does not give information about the toxic profile of a sample.^14^ Moreover, it is still considered a preliminary and complementary tool to chromatographic and mass spectrometry methods (LC-MS/MS).^15^ These analytical chemistry methods have strong potential, but they require special efforts to evidence the presence of CTXs, given the low limits that must be reached and the variety of analogues present in a sample.^16,17^

When these compounds are studied by LC-MS/MS methods, the use of solid-phase extractions (SPE) is almost mandatory given the complexity of the matrixes that contain the toxins.^14,17^ This is due to the fact that the raw extract obtained from fish samples still contains compounds (mainly lipophilic) that interfere with the ionization of CTXs. This matrix effect could act by either enhancing or suppressing the signal, with the latter being the worst-case scenario when working with low concentrations. Therefore, it is necessary to remove them, especially for the preparation of reference materials.^17–20^ While numerous studies using SPE cleanup procedures to elucidate the CTX profile of a sample have been carried out, few have been able to accomplish complete tests of the recovery, the matrix effect evaluation, and their efficiency.^17,19,21^ Usually, recovery and matrix effect studies are only performed with CTX1B^21^ or CTX3C^22^ by LC-MS/MS, and the results are applied to the quantification of the rest of the CTX analogues. This practice is common given the scarcity of standards for all analogues, the difficulty in obtaining different commercial standards, and also their high economic cost.^6,23–25^ Nevertheless, this assumption is not entirely precise since CTXs have a wide range of polarities. Thus, limited access to contaminated materials from other geographic areas and semipurified CTXs (in terms of economics and logistics) limits laboratory progress.^17^ The present study assesses the principal SPE protocols used by different research groups worldwide to evaluate CTXs in fish samples, providing information about recovery rates, matrix effects, and the efficiency of the cleanup procedure using CTX1B and CTX3C standards, as well as the interactions of C-CTX1 naturally present in the flesh matrix evaluated.

## 2 Material and Methods

### 2.1 Reagents and Standard Solutions

#### 2.1.1 Reagents and Solvents

Chemical reagents and solvents used during the sample extraction procedure and for the different cleanup steps were all of HPLC grade. Acetone, diethyl ether (DIEE), *n*-hexane, methanol (MeOH), acetonitrile (MeCN), ethyl acetate (AcOEt), acetic acid (HAc), and dimethyl sulfoxide (DMSO) were purchased from Honeywell; chloroform (CHCl_3_), dichloromethane (DCM), and 2-propanol were obtained from Fisher Chemical. Ultrapure water (resistivity >18 MΩ cm) was obtained using a Milli-Q water purification system (Millipore Ltd., Billerica, MA, USA).

For chromatographic analysis, all reagents were of LC-MS grade and from different providers. MeCN and deionized water were sourced from Supelco, MeOH and ammonium formate of LC-MS grade were purchased from Honeywell, and formic acid of LC-MS grade was obtained from Fisherbrand.

#### 2.1.2 Standard Solutions

Standard solutions used in the present research for recovery and matrix effect purposes CTX1B (1 μg) and CTX3C (1 μg) were provided by the Institute Louis Malardé (ILM, French Polynesia). The standard (STD) used as a reference for cytotoxicity assessment was CTX1B (2 μg) provided by R.J. Lewis (The Queensland University, Australia). The other standard solutions and reference materials for retention were sourced as follows: CTX3B (1 μg), 2,3-diOH-CTX3C (1 μg), M-seco-CTX3C (1 μg), and CTX4A (1 μg) were provided by the ILM; CTX2 (1 μg) and CTX3 (2 μg) were purchased from Dr R.J. Lewis; 51-OH-CTX3C (45.7 ng) was provided by Dr T. Yasumoto (Japan Food Research Laboratories (JFRL)); C-CTX3/4 (reference material for retention time confirmation only) was kindly ceded by Dr Alison Roberson (Dauphin Island Sea Lab, USA). All of them were dissolved in 1 mL of MeOH (LC-MS grade) and kept at −20 °C in glass vials.

### 2.2 Sample Treatment and Extraction

#### 2.2.1 Sample Origin and Treatment

Fish samples analyzed in this study were provided by the Institute of Animal Health and Food Safety (IUSA) at the University of Las Palmas de Gran Canaria. The samples consisted of a pool of flesh from 19 fishes (70% of Seriola spp. and 30% of Ephinephelus marginatus) captured in Canary Islands waters (caught between 2015 and 2018), sourced from both professional and recreational fishing within the framework of the EuroCigua project; then, these samples were previously examined by CBA, separately.^26^ After the confirmation of CTX-like toxicity in each fish extract, all remaining flesh was homogenized using a mixer blender, and three samples from the pooled flesh were extracted and assessed by CBA in order to verify the homogeneity of CTX-like toxicity across all homogenized flesh. Then, pooled flesh and one extract were kept at −20 °C until they were sent to IRTA facilities for analysis by LC-MS/MS. The presence of C-CTX1 was determined as the main analogue in the pooled flesh.^27^

#### 2.2.2 Sample Extraction

Toxin extraction for this pool was carried out following the protocol proposed by Yogi et al. (2011),^28^ with minor modifications described by Tudó et al. (2022).^29^ For the proposed experiment, 24 extracts were obtained. The following lines describe the extraction process for one extract in brief. First, 10 g of pool flesh was cooked at 70 °C for 10 min. Then, each sample was extracted twice with 2 mL of acetone per gram of tissue and homogenized using an Ultra-Turrax (T 25 basic IKA) at 17,500 × *g*. Acetonic extracts were recovered using centrifugation at 4 °C and 3000 × *g* for 10 min. Both supernatants of each sample were pooled and kept at −20 °C overnight. The next day, acetonic extracts were filtered with positive pressure through a 0.22 μm PTFE filter using a sterile syringe, before being dried out in a rotary evaporator at 60 °C; then, the resulting residue was recovered using 4 mL of deionized water and 16 mL of diethyl ether (DIEE) to perform a liquid–liquid partition twice in glass tubes. DIEE fractions were recovered with glass Pasteur pipettes. DIEE fractions were mixed and taken to dryness in a rotary evaporator at 60 °C. The residue was recovered with 2 mL of MeOH-H_2_O (8:2, *v/v*) and 4 mL of *n*-hexane in glass tubes. This second liquid/liquid partition was performed twice, where *n*-hexane layers were discarded using glass Pasteur pipettes. The phase with MeOH-water (8:2, *v/v*) was dried under a N_2_ current at 50 °C and then resuspended in 4 mL of MeOH.

With the aim of reducing the variability caused by slight differences during the extraction of each sample, the 24 methanolic extracts were pooled.

### 2.3 Sample Preparation

In order to evaluate the efficiency (eq 1) of the different clean-ups selected for the different analogues of CTXs chosen (CTX1B and CTX3C), the pooled extract divided into two large aliquots for recovery and matrix effect evaluation. The workflow schematic is shown in Figure S1.

1 $$\mathrm{SPE efficiency}=\frac{\mathrm{Recovery}\;\left(\mathrm{eq}\;2\right)}{\mathrm{Matrix Effect}\;\left(\mathrm{eq}\;3\right)}\times 100\%$$

#### 2.3.1 Recovery Assessment

For recovery assessment (eq 2), an aliquot of 44 mL of extract was spiked with 1.43 ng CTX1B/mL and 1.43 ng CTX3C/mL (these concentrations were chosen to obtain theoretical concentrations of 10 ng/mL after cleanup for both CTX1B and CTX3C). Then, 12 aliquots of 3.5 mL (i.e., 5 ng of CTX1B and 5 ng of CTX3C) were prepared (2 aliquots/SPE strategy) and evaporated to dryness under a N_2_ current to be resuspended in each SPE charge solvent.

2 $$\mathrm{Recovery}=\frac{\mathrm{STD concentration measured}}{\mathrm{STD concentration spiked}}\times 100\%$$

“STD concentration spiked” corresponded to the theoretical concentration in the final extract of each spiked sample (500 μL) before SPE (i.e., 10 ng/mL), assuming 100% recovery and no matrix effects during LC-MS/MS analysis. The mean of the individual recoveries was used for this evaluation.

#### 2.3.2 Matrix Effect Assessment

In order to evaluate the matrix effect (eq 3) in CTX1B and CTX3C standards, another 12 aliquots of 3.5 mL were prepared (2 aliquots/cleanup strategy) from the pooled extract and evaporated under a N_2_ current, and then, they were resuspended in the corresponding SPE charge solvent. After the respective SPEs, and in order to minimize the cost of the trial, 125 μL of each final extract was spiked with 10 ng CTX1B/mL and 10 ng CTX3C/mL. The remaining 375 μL of nonspiked extracts were kept for CBA analysis (see Section 2.5).

3 $$\mathrm{Matrix effect}=\frac{\mathrm{STD concentration measured}}{\mathrm{STD concentration spiked}}\times 100\%$$

“STD concentration spiked” corresponded to the theoretical concentration in the final extract spiked after SPE (10 ng/mL). The mean of the individual matrix effects was used for this evaluation.

### 2.4 Solid Phase Extractions (SPEs)

The SPE strategies selected for comparison in this study were chosen because they have been successfully used for one or more CTXs in different publications by different research groups.^2,17,19,21,22,28,30–34^

The performance of each cleanup protocol was evaluated in duplicate for the recovery and matrix effect. Additionally, each original SPE protocol was modified regarding tissue equivalents (TEs) used, in order to allow for the comparison of each strategy. Thus, all SPE strategies started from 3.5 mL of the pooled extract (2.5 g TE/mL) and were taken to a final volume of 500 μL (17.5 g TE/mL).

For all SPE protocols, the final fractions (*F*_n_) (washes and elutes) were reduced to dryness under a N_2_ stream at 50 °C, reconstituted with 500 μL of MeOH twice, and filtered through a 0.22 μm PTFE filter (FILTER-LAB 4 mm Whatman Puradisc) into a glass vial. Then, to minimize toxin losses, another 500 μL of fresh MeOH was passed through the used filters and pooled in the corresponding vial. Afterward, the cleaned extract (∼1500 μL) was dried out under a N_2_ flow at 50 °C to be finally reconstituted in 500 μL of MeOH and kept at −20 °C until further steps and analysis.

#### 2.4.1 First Strategy (Florisil-C18)

This purification protocol was followed as described by Estevez et al. (2019)^21^ and was based on the previous methodology of Yogi et al. (2011)^28^ and Sibat et al. (2018),^30^ with slight modifications. The cartridges chosen for this strategy were Florisil (J.T. Baker, 500 mg/3 mL) and Octadecyl (C18) (J.T. Baker, 500 mg/3 mL). First, dried samples were reconstituted with 3 mL of AcOEt, then loaded to the normal-phase SPE cartridge (Florisil) previously conditioned with 3 mL of AcOEt, and washed with 3 mL of AcOEt (first SPE fraction, F1). Subsequently, toxins were eluted with 2 × 2.5 mL of AcOEt-MeOH (9:1, *v*/*v*) and 2 × 2.5 mL of AcOEt-MeOH (3:1, *v*/*v*); both eluates were collected as the same fraction (eluate SPE fraction, F2). Normal-phase SPE was carried out at a flow rate of 0.5 mL/min. F1 and F2 were dried out under a N_2_ stream; then, F1 was reconstituted in 500 μL of MeOH as previously mentioned (see Section 2.4), and F2 was reconstituted with 2 mL of MeOH-H_2_O (6:4, *v*/*v*).

The reverse-phase SPE cartridge (C18) was previously conditioned with 3 mL of MeOH-H_2_O (6:4, *v*/*v*); then, the reconstituted F2 was loaded onto the cartridge, washed with 4 × 2.5 mL of MeOH-H_2_O (6:4, *v*/*v*), and collected as F3. Afterward, toxins were eluted with MeOH-H_2_O (9:1, *v*/*v*) as F4. An additional eluate was collected using 100% MeOH and classified as F5. Reverse-phase SPE was carried out at an approximate flow rate of 1 mL/min. Reverse-phase fractions (F3, F4, and F5) were dried under a N_2_ stream and reconstituted in 500 μL of MeOH. Ciguatoxins are expected in F4 and F5.^21^

#### 2.4.2 Second Strategy (Florisil)

The Florisil SPE protocol was performed according to the descriptions by Murata and Yasumoto (2019)^22^ and Estevez et al. (2023).^2^ The dried extract was reconstituted with 2 mL of *n*-hexane-acetone (4:1, *v*/*v*) and loaded to a normal-phase SPE Florisil cartridge (J.T. Baker, 500 mg/3 mL), previously conditioned with 3 mL of *n*-hexane-acetone (4:1, *v*/*v*). The sample was washed with 3 mL of *n*-hexane-acetone (4:1, *v*/*v*) at a flow rate of 0.6 mL/min and collected as F1. Afterward, toxins were eluted with 3 mL of acetone-MeOH (9:1, *v*/*v*) at a flow rate of 1 mL/min. Then, the eluted fraction was identified as F2. Both fractions (F1 and F2) were dried under N_2_ stream and reconstituted in 500 μL of MeOH.

#### 2.4.3 Third Strategy (P.DVB-Silica)

The third strategy was carried out as proposed by Spielmeyer et al. (2021),^17^ which was adapted from Nagae et al. (2021)^31^ and Lewis et al. (2009),^19^ with minor modifications according to our laboratory needs. All SPE steps were performed without a vacuum, except when the remainder of the column was removed. The dried aliquots were resuspended in 5 mL of MeOH-H_2_O (8:2, *v*/*v*) and loaded to the reverse-phase SPE polar-modified polystyrene-divinylbenzene (P.DVB) copolymer cartridge (Macherey-Nagel CHROMABOND EASY, 200 mg/3 mL) previously conditioned with 3 mL of AcOEt (0.1% acetic acid (HAc)), 2 × 3 mL of MeCN, and 3 × 3 mL of MeOH-H_2_O (8:2, *v*/*v*). The glass tube containing the sample was rinsed with 2 × 1 mL of MeOH-H_2_O (8:2, *v*/*v*) and was used to wash the cartridge. The liquid retained by the column was also collected; therefore, the resulting fraction was identified as F1. The toxins trapped in the P.DVB cartridge were eluted using 3 mL of MeCN and 2 × 2.5 mL of AcOEt (0.1% Hac); the remaining solvents in the column were also recovered and mixed with the eluate identified as F2.

For the normal-phase SPE, F2 was dried out under a N_2_ stream and then resuspended in 2 mL of *N*-hexane to be loaded to a silica cartridge (Agilent HF Bond Elut-SI, 500 mg/3 mL), preconditioned with 3 mL of AcOEt (0.1% HAc)-MeOH (3:1, *v*/*v*), 2 × 3 mL of AcOEt (0.1% HAc), and 3 × 3 mL of *n*-hexane-AcOEt (0.1% HAc) (1:1, *v*/*v*). As in the reverse-phase SPE, a glass tube was rinsed with 2 × 1 mL of *n*-hexane-AcOEt (0.1% HAc) (1:1, *v*/*v*) and used to wash the cartridge. Afterward, 1 mL of *n*-hexane-AcOEt (0.1% HAc) (1:1, *v*/*v*) was added to the column. All these filtered volumes were recovered and identified as F3. Toxins retained by the silica cartridge were eluted using 3 mL of AcOEt (0.1% HAc), followed by 2 × 2.5 mL + 2 mL of AcOEt (0.1% HAc)-MeOH (3:1, *v*/*v*), and the retained liquid in the column was recovered and pooled with the eluted fraction named F4. All fractions obtained were dried out under N_2_ current and resuspended in 500 μL of MeOH.

#### 2.4.4 Fourth Strategy (Amino)

This protocol was followed as described by Estévez et al. (2021)^32^ to remove phosphatidylcholine from amberjack samples. This protocol was also proposed by Alvarez and Touchstone (1992)^35^ for other applications. The sample extract was dissolved in 300 μL of CHCl_3_ and loaded into an aminopropyl cartridge (Supelco Supelclean LC-NH_2_, 500 mg/3 mL), which was previously conditioned with 3 mL of *n*-hexane. The sample was eluted using 5 mL of 2-propanol/CHCl_3_ (1:2, *v*/*v*) at a flow rate of 1 mL/min. The only fraction recovered was identified as F1, dried out, and resuspended in 500 μL of MeOH.

#### 2.4.5 Fifth Strategy (Silica)

Silica SPE was performed as described by Kryuchkov et al. (2022).^33^ The dried sample was reconstituted with 2 mL of DCM and loaded to a silica cartridge (Agilent HF Bond Elut- SI, 500 mg/3 mL) preconditioned with 3 × 3 mL of MeOH and 3 × 3 mL of DCM. The glass sample tube was rinsed with 3 × 500 μL of DCM, and these volumes were used to wash the SPE cartridge. Additionally, 2 × 3 mL of DCM were added to the silica column and collected as F1. Toxins present in the sample were eluted with 3 × 3 mL of MeOH/DCM (1:9, *v*/*v*) and identified as F2. No vacuum was used through the different steps.

#### 2.4.6 Sixth Strategy (Flo-Amino)

The cleanup was carried out following the protocol indicated by Oshiro et al. (2022).^34^ A dried aliquot of the sample extract was redissolved in 5 mL of AcOEt-MeOH (9:1, *v*/*v*) and loaded into a SPE Florisil cartridge (J.T. Baker, 500 mg/3 mL) previously conditioned with 2 × 2.5 mL of AcOEt-MeOH (9:1, *v*/*v*). Once the loaded sample was eluted (F1) from the cartridge at a flow rate of 0.85 mL/min, it was evaporated to dryness under a N_2_ stream at 40 °C and reconstituted in 5 mL of MeCN.

Redissolved F1 was then loaded to an aminopropyl cartridge (Supelco Supelclean LC-NH_2_, 500 mg, 3 mL), preconditioned with 3 mL of MeCN and 3 mL of MeOH. The eluted sample was recovered as F2, and a second elution was performed with 3 mL of MeOH and identified as F3. Both fractions are expected to contain CTXs, according to the literature. All the steps for the aminopropyl cartridge were performed at a flow rate of 1 mL/min.

### 2.5 N2a Cell-Based Assay (CBA)

For evaluation by cell-based assay (CBA), one aliquot of the initial pooled extract before SPEs was prepared (F0), and 375 μL from each of the remaining matrix effect assessment fractions (not spiked) (see Section 2.3.2 and Figure S1) were analyzed at the same time, on two different days (two replicates), in order to evaluate the toxicity recoveries from each SPE fraction in relatiin to the CTXs naturally present in the pooled flesh.

Neuroblastoma cells (Neuro-2a cells: CCL-131, from ATCC, LGC Standards SLU, Barcelona, Spain) were maintained in the Roswell Park Memorial Institute (RPMI)-1640 medium supplemented with 10% fetal bovine serum (FBS) at 37 °C under a 5% CO_2_ atmosphere.

The CBA was carried out as proposed in Caillaud et al. (2012)^36^ with minor modifications. For this task, an eight-point dose–response curve was performed for each sample. Briefly, cells were seeded in a 96-well flat-bottom microplate at a concentration of 34,000 cells/well in the RPMI medium supplemented with 5% FBS. After 24 h of incubation, half of the microplate was treated with 0.13 mM ouabain and 0.013 mM veratridine in order to assess CTX-like cell mortality when exposed to samples or STD at decreasing concentrations. 20 h later, cell viability was estimated using the MTT assay [3-(4,5-dimethylthiazol-2-yl)-2,5-diphenyltetrazolium] and DMSO. Microplates were read with a multiwell spectrophotometer scanner (Agilent BioTek Synergy LX multimode reader) at 570 nm, and data were analyzed with Microsoft Office Excel 2021 and GraphPad Prism 9 software (GraphPad, San Diego, CA, USA).

All samples were exposed starting at 300 mg of tissue equivalents (TE)/mL with 7 serial dilutions (1/2) to additionally assess the matrix effect resulting from SPE strategies. Determination of CTX-like toxicity of each sample was performed by comparison with the STD curve (IC_50_ = 0.799 ± 0.062 pg CTX1B/mL) from the corresponding day assay. The LOD/LOQ was established according to the IC_20_ from the STD (0.364 ± 0.053 pg CTX1B/mL) and by considering the maximum concentration (max: 300 mg TE/mL) of sample extracts (*n* = 12 plates) exposed with nonspecific toxicity (0.0012 ± 0.0002 pg eq CTX1B/g TE of flesh). Besides, a cell mortality below 20% was considered as a nontoxic effect.

### 2.6 Liquid Chromatography-Tandem Mass Spectrometry (LC-MS/MS) Analysis

#### 2.6.1 LC-MS/MS Instrument

For LC-MS/MS analysis, a Xevo TQ-XS (Waters Corporation, Milford, MA, USA) coupled to an Acquity UPLC I-Plus-Class (Waters Corporation, Milford, MA, USA) was utilized. Nitrogen supply was provided by a generator NM20Z (Peak Scientific, Renfrewshire, Scotland, UK). Instrument control, acquisition, and data analysis were powered by MassLynx V4.2, TargetLynx XS software (Waters Corporation, Milford, MA, USA), and Microsoft Office Excel 2021.

#### 2.6.2 Liquid Chromatography Method

Analytical separation of the compounds presented in the SPE fractions, and the pooled extract was performed according to Tudó et al. (2022),^29^ with some modifications. Briefly, an Acquity Premier BEH C18 column (50 mm × 2.1 mm, 1.7 μm particle size, Waters Corporation, Milford, MA, USA) was used at 40 °C, and a binary gradient elution was carried out with mobile phase A (H_2_O + 2 mM ammonium formate + 0.1% formic acid) and mobile phase B (MeCN + 5% H_2_O + 2 mM ammonium formate + 0.1% formic acid).

The gradient elution flow rate was set at 0.4 mL/min, the injection volume was 2 μL, and each sample was injected twice. The gradient description is further detailed in Table S1

Additionally, the matrix effect was evaluated using different injection volumes to determine the impact of volume on the outcome. Two microliters (2 μL) and five microliters (5 μL) were compared.

#### 2.6.3 Mass Spectrometer Analytic Method

The source of the mass spectrometer utilized was an ESI in positive ion mode, with a capillary voltage of 3.0 kV, a source temperature of 150 °C, a desolvation temperature of 450 °C, N_2_ gas flow for desolvation at 1000 L/h, cone gas flow at 300 L/h, and nebulizer gas flow at 7 bar. The collision cell was operated with 0.15 mL/min argon.

Multiple reaction monitoring modes (MRMs) were performed to assess the following CTX analogues (ordered according to the retention times from standards and internal reference materials): C-CTX3/4; 17-OH-C-CTX1; CTX1B; C-CTX1; M-seco-CTX3C; CTX2; 2,3-diOH-CTX3C; 51-OH-CTX3C; CTX3; CTX3B; CTX3C; CTX4A (for details of transitions monitored, quantization and confirmation signals, and also energies applied to each CTX congener, see Table S1). All of them were evaluated in the original pooled sample extract provided by the IUSA laboratory^27^ prior to the spiking experiments conducted in this study. Only a clear signal for C-CTX1 and possible traces of 17-OH-C-CTX1 were detected. Therefore, CTX1B, CTX3C, C-CTX1, and 17-OH-C-CTX1 were then monitored in this study. At least two MRM transitions different from the sodium adduct were monitored for every analogue, with a dwell time of 0.3 ms per transition. Identification was supported by toxin retention time and MRM ion ratios.

Quantification of CTX signals was carried out with CTX1B for the CTX4A group, the C-CTX group, and CTX3C for the CTX3C group toxins. For the CTX1B standard, a nine-level calibration curve was obtained (0.08–28 ng CTX1B/mL), showing a good intrabatch performance and linear adjustment with *R* ^2^ ≥ 0.999. The deviation of the slopes between consecutive scans was less than 14%. For sample extracts, the LOQs were 0.032 and 0.0046 ng CTX1B/g TE before and after SPE, respectively. In the case of CTX3C, a six-level calibration line was obtained (0.5–28 ng CTX3C/mL) with *R* ^2^ ≥ 0.991, a deviation below 22%, and LOQs of 0.2–0.028 ng CTX3C/g TE for sample extracts before and after SPE, respectively.

## 3 Results and Discussion

### 3.1 Evaluation of Recovery of CTX-like Toxicity by CBA

Usually, methanolic extracts from fish suspected of containing CTXs are evaluated by CBA as a previous step before performing solid-phase extraction (SPE) for chromatographic analysis and confirmation. However, no information about the toxicity recovery is provided after the cleanup process, making it difficult to evaluate losses of known and unknown CTXs.^20,37^ When this information is provided, it is relative to recoveries or matrix effects normally assessed by LC-MS/MS rather than by CBA, and it is usually related to the CTX1B standard. It is noteworthy that few research groups can afford the simultaneous use of both standards (CTX1B and CTX3C), or even more, for multiple samples,^17,30^ due to the high cost of the scarce commercially available standards.

To study the efficiency of SPEs about the toxicity recovery in relation to known and unknown CTXs, both, the original extract from pooled flesh before SPE (referred in this study as F0) and every fraction resulted from the different steps of the clean-ups (*F*_n_ non-spiked) (for more details, see Section 2.3.2), were assessed by CBA to measure the toxic potential of the naturally contained toxins in the flesh pool. The resulting CTX-like toxicities and percentages of the recovered toxicity by each SPE strategy (*F*_n_) as determined by CBA are summarized in Table 1.

**Table 1:** Estimated Toxicity of Each SPE Fraction (*F*_n_) Assessed by CBA and Expressed as ng of CTX1B eq/g Equivalent of Flesh

SPE strategy (acronym)	Fraction (*F*_n_)	CTX-like toxicity (ng eq CTX1B/g TE)	SD (*n* = 2)^a^ ^a^	Toxicity recovered from F0^b^ ^b^
No SPE	0	0.109	0.0027	-
1st Florisil-C18 (Flo-C18)	1	<LOD	-	-
	3	<LOD	-	-
	4	0.014	0.0014	12.5%
	5	<LOD	-	-
2nd Florisil	1	0.003	0.0002	2.4%
	2	0.015	0.0005	14%
3rd P.Divinylbenzene-Silica (P.DVB-Silica)	1	<LOD	-	-
	3	<LOD	-	-
	4	0.058	0.0094	53.3%
4th Aminopropyl (Amino)	1	0.012	0.0032	11.4%
5th Silica	1	<LOD	-	-
	2	0.090	0.0324	82.3%
6th Florisil-aminopropyl (Flo-Amino)	2	<LOD	-	-
	3	0.005	0.0009	4.7%

a Two measurements were taken using data from three wells for each exposure concentration per measurement.

b Toxicity recovery estimated using F0 as 100% of toxicity. SD: standard deviation.

All the different cleanup strategies were able to recover fractions with CTX-like toxicity from the naturally contaminated flesh extract, but at different degrees. Flo-Amino SPE recovered fractions with the lowest toxicity, which indicates a near-total loss of the CTX-like compounds present in the original extract. This result was followed by Amino SPE and Flo-C18 SPE with similar toxicity recoveries. Florisil cleanup was able to recover fractions with higher toxicity compared to the previous SPE strategies. The percentage of estimated toxicity from the initial toxicity was calculated by considering both fractions obtained during the SPE (F1 = 2.4% + F2 = 14.0% for Florisil). The P.DVB-Silica SPE recovered fractions with higher toxicities, more than half of the original toxicity. Similar results were obtained by Loeffler and Spielmeyer (2024)^38^ with an approximate 40% loss of toxicity in different matrices. The results of the CBA were in contrast with the good recoveries for CTX standards spiked for chromatographic assessment (see Section 3.2.4). Perhaps, the naturally occurring CTX compounds (unknowns) present in the extract analyzed here were not released from the SPE cartridges with the solvents used, or the acidification of the sample favored the changes of nonmajor analytes.^38^ Finally, silica SPE resulted in fractions with more than 80% of the initial toxicity, making this method and the P.DVB-Silica SPE the best cleanup strategies for the CBA. However, in the silica SPE, the fraction that contains the toxins (F2) showed a nonspecific toxicity above 150 mg TE/mL, maybe due to some matrix compounds, but with an unusual profile compared to the CBA of F0 (Figure 1). In this case, the cell viability in wells pretreated with (O/V) was higher at 150 and 300 mg TE/mL than that at 75 mg TE/mL. Therefore, Silica SPE was the only cleanup strategy that was not able to remove interferences from the matrix. This could result in a false negative when samples are analyzed at high tissue equivalents (i.e., screening method of two dilution points; see Figure 1). This fact must be taken into consideration if silica SPEs are intended to be introduced in routine for monitoring programs that carry out sample analysis through CBA screening at relatively high TE, like the one carried out in the Canary Islands.^10,11^

**Figure 1:**
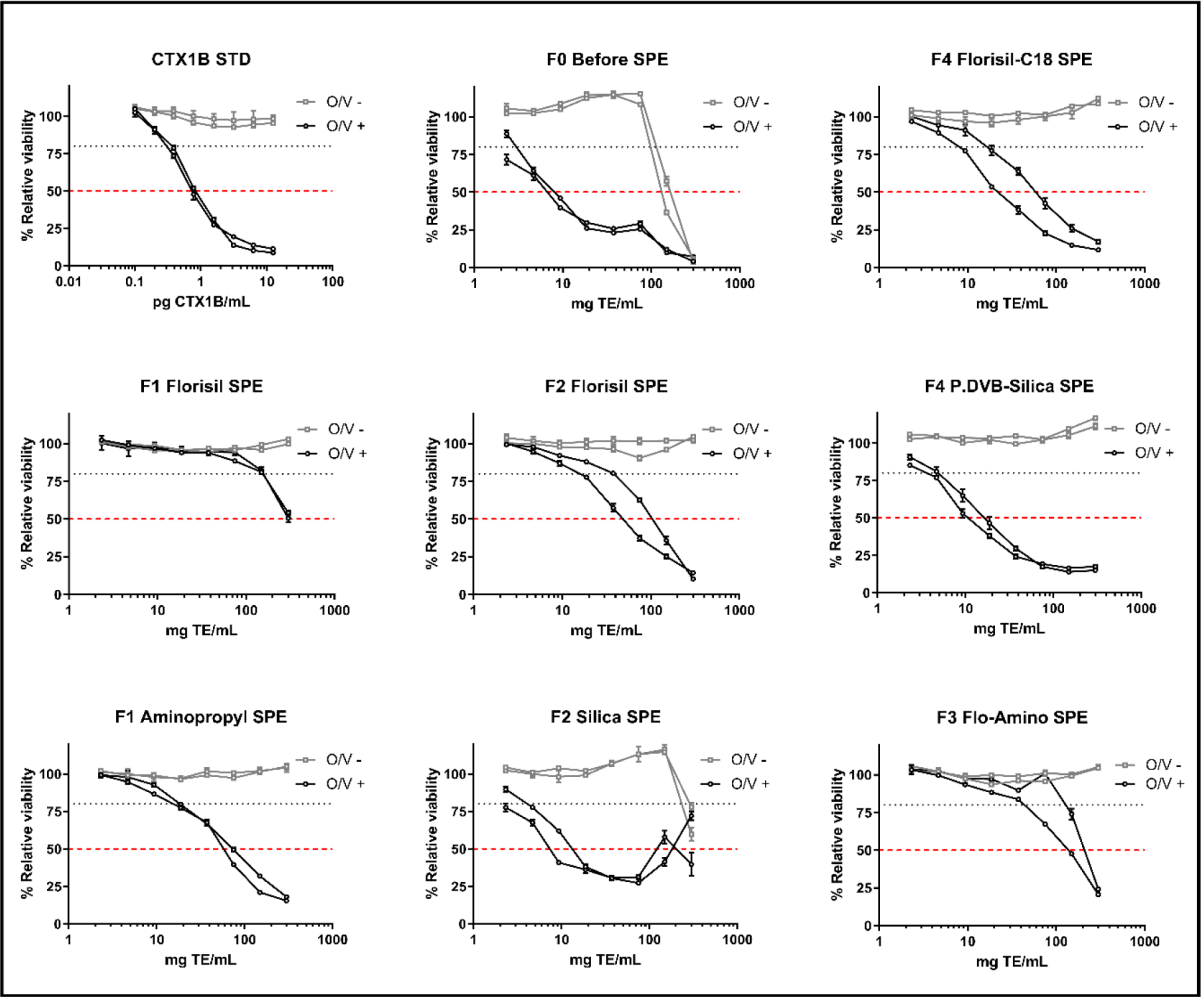
Dose–response curves of CTX1B standard solution, raw flesh extract (F0), and each SPE fraction (*F*_n_) with measurable toxicity. The superimposed graphs correspond to analyses of the same sample on different days.

### 3.2 Evaluation of Matrix Effects and Recovery by LC-MS/MS

To characterize the toxin profile and to evaluate the efficiency of the different clean-ups in the different fractions previously analyzed by CBA, LC-MS/MS analyses were performed.

Chromatographic performance of the different fractions obtained during the SPE steps is given in percentages of CTX1B and CTX3C and is included in Figure 2 and Table S3. For C-CTX1, due to the lack of a commercial standard, only data of chromatographic areas in ng eq of CTX1B/mL are reported and compared in the following sections.

**Figure 2:**
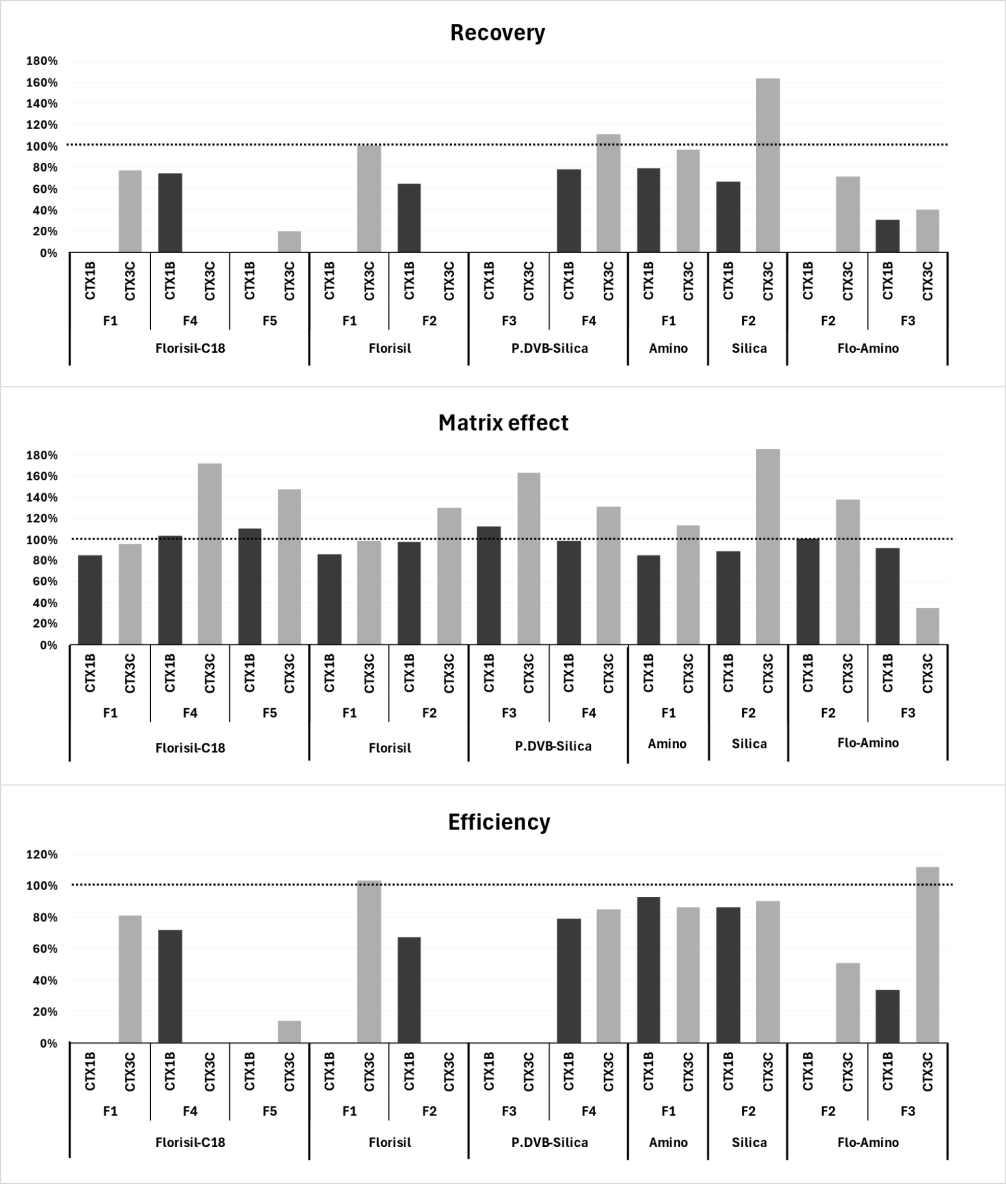
Recovery (RE), matrix effect (ME) and efficiency for CTX1B and CTX3C in the different SPEs spiked at 10 ng/mL expressed as % in the different fractions (for further details, see Table S3).

Given the differences between the clean-ups evaluated and the possibility that one or two toxins appeared in two or more fractions of the same SPE, as demonstrated by the CBA analysis, all fractions (*F*_n_) with measurable CTX-like toxicity were spiked at 10 ng/mL of CTX1B and CTX3C to assess the matrix effect, as well as F5 from Florisil-C18, F3 from P.DVB-Silica, and F2 from Flo-Amino clean-ups (for more details, see Section 2.3.2). All results are summarized in Figure 2 and Table S3.

#### 3.2.1 First Strategy (Florisil-C18)

The protocol followed here was carried out by Sibat et al. (2018)^30^ for Pacific CTXs in snail (Tectus niloticus), sea urchin (Tripneustes gratilla), parrotfish (Chlorurus microrhinos), groupers (Epinephelus polyphekadion), and Gambierdiscus polynesiensis matrices, and was used by Estevez et al. (2019)^21^ for C-CTX1 in amberjack samples. This cleanup was an adaptation of the one described by Yogi et al. (2011)^28^ with acceptable results in different matrices such as different invertebrates and fish tissues. This strategy consists of two cleanups and two different cartridges, first the Florisil and then the C18, generating four fractions. F1 refers to the filtrate obtained during Florisil SPE, F3 is the other filtrate fraction but obtained from the C18 SPE, and finally, F4 and F5 are considered as eluates of the C18 cartridge where CTXs may appear according to the references. In this study, CTX1B and C-CTX1 appear exclusively in F4. This fraction gives a matrix effect (3% enhancement) and a good recovery for CTX1B, resulting in an SPE efficiency of 72%. The result obtained contrasts with the only data available in this regard, given by Estevez et al. (2019),^21^ where they obtained a 57.6% recovery for CTX1B. Besides, the matrix effect was quite similar to data reported by Sibat et al. (2018), about 85–115% in the CTX1B signal monitored by ammonium and protonated adducts; however, no experiments about recoveries were performed at that time .

Regarding CTX3C, the transition signals were detected in fractions F1, F4, and F5; however, the signals detected in F4 were ruled out by ion ratio relation (>35% difference). It is important to note that almost 80% of the CTX3C spiked before SPE appeared in the F1 considered as the filtrate or waste and, therefore, a fraction that is not usually evaluated. This result demonstrates the low capacity of the Florisil cartridge used in this experiment to retain the most polar CTXs. The small amount of toxin retained during the washing step, about 14% of the CTX3C, was released in F5 when 100% MeOH was passed through the column. Interestingly, the F1 fraction exhibited a low matrix effect (4% ion suppression). This value for the signal monitored for CTX3C in F5 was determined as 48% enhancement.

In relation to C-CTX1, its chromatographic result was among the best in this study (Table S3). However, the toxicity results by CBA demonstrated the loss of other toxic compounds naturally present in the sample (Table 1). Moreover, Octadecyl (C18) cartridges are increasingly out of use for the study of CTXs in favor of other reversed-phase cartridges,^17,39,40^

#### 3.2.2 Second Strategy (Florisil)

This SPE protocol was considered in this trial because of the good results obtained by Estevez et al. (2023)^2,41^ for C-CTX1 in amberjack flesh. This cleanup was also carried out by Murata and Yasumoto (2019)^22^ and considered suitable for the assessment of CTX3C and brevetoxins by the receptor binding assay (RBA) in parrotfish and grouper tissues. From this strategy, two fractions were collected (F1, filtrate and F2, eluate). Although the CTX1B, C-CTX1, and CTX3C were able to be detected by LC-MS/MS, not all of them were recovered in the considered eluate fraction (F2). CTX1B was recovered in F2, C-CTX1 was detected in F1 and F2 in a ratio of 1:3, and CTX3C was found only in F1. Even with this distribution in the CTX analogues, the resulting matrix effect was very low, about 2% in F2 and 1% in F1 of ion suppression in CTX1B and CTX3C signals, respectively. However, recovery rates were very different, CTX1B recovery only reached 65% of the spiked analogue, and for CTX3C, all analyte was recovered (103%) in F1. This finding supports the result obtained in the Flo-C18 strategy, demonstrating that the Florisil cartridge was not able to retain this analogue even when different solvents were used. This situation, combined with the fact that C-CTX1 was also found in a low proportion in F1 (just under LOQ) (Table S3), suggests that the Florisil cartridge used could not retain 100% of the toxins with a polarity lower than CTX1B (according to the retention times).

Results of some preliminary tests conducted in this laboratory using this SPE strategy, under the same conditions, in different fish samples – for instance, cubera snapper (Lutjanus cyanopterus), moray eel (Muraena augusti), and amberjack (Seriola spp.) flesh with C-CTX1 naturally present, and other non-ciguatoxic amberjack flesh spiked with standard solutions of CTX1B and CTX3C (Figure S2) – were completely different. In those previous analysis, all CTXs were eluted in F2 with excellent recoveries (91% and 139%) and matrix effects (88%% and 120%) for CTX1B and CTX3C, respectively. Additionally, all C-CTX1 signals were detected only in F2 (even other C-CTX-related analytes more polar than CTX1B), making this a promising SPE strategy that needed to be evaluated comparatively with other cleaning strategies. These preliminary studies were carried out in in this laboratory until the necessary amount of flesh to perform this study was available. When that occurred, the batch of Florisil cartridges used previously was finished, and a new different batch began to be used. Additionally, the unexpected result for the performance of CTX3C in Flo-C18 SPE could be also due to the change of the batch of the Florisil cartridges. This situation regarding Florisil batches is similar to that experienced by Nagae et al. in 2021 ^31^ and coincides with the knowledge of the fluctuation in the quality of Florisil cartridges (Schenk et al., 1996).^42^ This fact implies that all SPE strategies that use Florisil cartridges are dependent on the quality of the lot that is available at a given time; therefore, it would be necessary to evaluate the performance of standard compounds in each Florisil lot before using samples, which is cost-prohibitive for a regular CTX analysis.

#### 3.2.3 Third Strategy (P.DVB-Silica)

This cleanup strategy was proposed by Spielmeyer et al. (2021),^17^ and it arose mainly from the protocol described by Nagae et al. (2021)^31^ and Lewis (2009)^19^ that uses two SPE in tandem: first, a reverse-phase SPE (C18), substituted with a polar-modified P.DVB copolymer cartridge, and then a normal-phase SPE (Silica). There were three fractions resulting from this strategy (F1 filtrate, F3 filtrate, and F4 eluate). F1 was not injected due to the large amount of suspended particles it contained. Even with this inconvenience, the LC-MS/MS analysis demonstrated that all CTXs used in this study were recovered in F4.

The CTX1B recovery was nearly 80% and a negligible matrix effect (1% signal suppression), which was one of the best results in this research study for this compound. This finding cannot be compared to the recovery rate reported by proposers, because they spiked CTX1B directly into the flesh before extraction. Their results, according to ion suppression, ranged from 0% to 46% in the fish species studied (Epinephelus areolatus, Scarus ghobban, and Lutjanus malabaricus) when sodium adducts were monitored. In this study, sodium adducts were not considered good comparative points between SPEs given the evident matrix effect, when they were monitored under the chromatographic conditions applied (see Figures S2 and S3).

The CTX3C result for this SPE obtained an 85% efficiency in F4. This result contrasts with the previous results reported by Spielmeyer et al. (2021).^17^ That group found that CTX3C was split up between the filtrate (F3) and the eluate (F4) at a ratio of 2:3 in all matrices used. Additionally, medium-low polar CTXs (CTX2 and CTX3) were found in low concentrations in F3. The most significant discrepancy between the steps followed by the proposers and the ones followed in the present research was that the resulting eluate (MeCN and AcOEt (0.1% HAc)) from reverse-phase SPE was not reduced to 2 mL to be mixed with 2 mL of *n*-hexane before loading into the silica cartridge (for more details, see Section 2.4.3). Therefore, the filtrate (F2) from P.DVB SPE was dried out under a N_2_ stream and then resuspended only in 2 mL of *n*-hexane. This reduced volume may have prevented the split up of CTX3C between the filtrate (F3) and eluate (F4), since large volumes of samples could lead to greater transfer of CTX3C into the filtrate fraction (F3), as suggested by the authors of this cleanup strategy.^17^ However, the absence of MeCN in the sample before loading into the silica column could also affect this result. Since, in the framework of our research, a small trial simulating this step using only *n*-hexane (without MeCN) demonstrated that this solvent itself is not capable of dissolving any of the CTXs from a dried glass tube and it is the second step (*n*-hexane-AcOEt (0.1% HAc) (1:1, *v*/*v*)) responsible for transferring CTXs from the glass tube to the silica cartridge, with recoveries near 100%, it is possible to skip the *n*-hexane step and go straight the to *n*-hexane-AcOEt (0.1% HAc) (1:1, *v*/*v*) step.

Regarding C-CTX1, this SPE protocol obtained the highest chromatographic areas for the signals monitored for C-CTX1 (Table S3), which is in accordance with the CBA results, but it also suggests that a portion of the nonmajor toxic analytes was lost (Table 1).

#### 3.2.4 Fourth Strategy (Amino)

The aminopropyl cleanup is actually a part of a SPE described by Alvarez and Touchstone (1992)^35^ for lipid isolation. The aim of this strategy is to remove the nonpolar lipids such as cholesterol esters, triglycerides, and diglycerides from the sample. Thus, this simple SPE was proposed by Estévez et al. (2021)^32^ to remove phosphatidylcholine from amberjack samples, given the strong ion suppression in the C-CTX1 signal found during their research. The recovery then reported (89%) was only assessed in a CTX1B pure standard passed through the SPE cartridge, and this data was directly applied to their C-CTX1 quantifications. Despite the differences between their procedure and the ones carried out here, the results obtained in this research were similar: 79% recovery in the spiked flesh sample and 85% matrix effect, which results in a SPE efficiency of 93% for this analogue.

Regarding CTX3C, recovery and matrix effect were also quite good, achieving 97% and 113%, respectively, with an efficiency of 86% for this toxin. For C-CTX1, the chromatographic area measured was similar to the Flo-C18 cleanup, immediately following the P.DVB-Silica strategy (Table S3). However, the CBA results again suggest the loss of a large part of the unknown toxic analytes present in the sample (Table 1).

#### 3.2.5 Fifth Strategy (Silica)

The silica SPE method is a fragment of the cleanup method proposed by Lewis (2009)^19^ and modified by Kryuchkov et al. (2022),^33^ in which the reverse-phase SPE has been ruled out, and the process goes directly to the normal-phase SPE using a silica column. The three analogues of CTXs studied were eluted in F2.

The cleanup efficiency for both standards spiked was quite promising (90% for CTX1B and CTX3C). However, in the nonpolar analyte (CTX3C), the result of the matrix effect was the highest among all the SPEs compared, with about 83% enhancement of the monitored signal. Previous studies using this SPE strategy do not allow for comparison because there is no reported recovery or matrix effects data in CTX1B and CTX3C standards, since it has been mainly used in the research of CTXs from the Caribbean region.^33,43,44^ Regarding C-CTX1, the signal area measured was slightly lower than that in the aminopropyl and Flo-C18 strategies (Table S3). This result could be influenced by the presence of coextractives, as suggested by the CBA results of this SPE, although the difference is minimal.

#### 3.2.6 Sixth Strategy (Flo-Amino)

The Florisil-aminopropyl cleanup was implemented as described by Oshiro et al. (2021)^34^ who referred efficiency data for CTX1B of 79–90% from Yogi et al. (2014).^45^ In this strategy, it has been described that CTXs eluted in two different fractions. Low-polar congeners (CTX4A, CTX4B, CTX3C, CTX3B, and 51-hydroxyCTX3C) eluate with MeCN (F2), and more polar analogues (CTX1B, CTX2, CTX3, 2,3-dihydroxyCTX3C, and 2,3,51-trihydroxyCTX3C) elute, when MeOH is used (F3).

For the SPE carried out in this study, CTX1B signal suppression was only 8% in F3. However, recovery was very low, about 31%, showing the worst result for this analogue in the present study, and was not in accordance with the results from Yogi et al. (2014).^45^ The CTX3C analogue was only expected in F2; nevertheless, this CTX was found in F2 and F3 in a ratio of 7:10. However, if both fractions are considered together, efficiency for this congener reached 86%. Regarding C-CTX1, the signal measured was the lowest in this research, except for the F1 of the second strategy (Florisil SPE) (Table S3), and therefore, the result of recovered CTX-like toxicity was very low (Table 1). These unexpected results could be influenced in part by the Florisil cartridge lot used.

#### 3.2.7 Analysis of Sodium Adducts

The sodium adduct transitions ([M + Na]^+^ > [M + Na]^+^), also known as the “transition trap”, were monitored in this study only for CTX1B and CTX3C. They were not considered for quantification purposes, but rather they were used to analyze their matrix effect performance of the different SPE carried out. In the case of CTX1B, it is worth mentioning that this transition was greatly suppressed in all SPE fractions analyzed, while in the standard solution, it was the most sensitive transition (under the conditions here conducted) (Figure S2). For CTX3C, it was completely opposite. All signals were enhanced, which also led in some cases to an increase of the signal–noise levels (Figure S4). These findings reinforce the need to perform SPE efficiency analysis in each study, especially in those that use methodologies favoring this *pseudotransition* to quantify CTX analogues and limiting the presence of confirmation transitions.

By comparison, the C-CTX1 sodium adduct was not monitored since in previous analyses this transition was not detected in most toxic samples (data not shown), nor in the sample used as a reference. This could suggest that the instrument and the conditions conducted here do not favor the formation of this adduct. It is also important to keep in mind that no pure standard solution of this compound is available to measure its performance under the analytical conditions used in this study, without matrix interferences. The CTXs naturally present in a sample could be attached to or influenced by interactions with coextractives that do not react with standards artificially added.

#### 3.2.8 Analyses of C-CTX1 Performance Using Different Injection Volumes

The matrix effect was also assessed for C-CTX1 by LC-MS/MS when different volumes (2 and 5 μL) were injected. For this task, the entire sequence was reinjected, obtaining two replicates for each *F*_n_ of SPE analyzed.

In general, responses were reduced by about 20% when 5 μL were injected (including CTX1B and CTX3C), except for F4 Flo-C18 and F2 Florisil, in which the response was similar to that obtained when using 2 μL. More remarkable was the fact that when more sample was injected, the signal of C-CTX1 in the extract before SPE (F0) was not detectable. These results reinforce how the coextractive compounds interfere with the C-CTX1 ionization, similar to what was observed in CTX1B.^39^

### 3.3 Comparative Overview

The availability of CTX standards is limited and cost-prohibitive for certain experiments.^6,23^ Therefore, CTX1B results after clean-ups are usually applied to other CTXs, such as C-CTX1.^2^,^21,32^ It makes sense to assume that the more similarity between the polarity of the analyzed CTX with the CTX1B, the more precise the calculation is, as is the case with C-CTX1 (RT min: 2.33), which has an elution time very close to CTX1B (RT min: 2.28) (Figure 3). However, the Florisil SPE results showed that C-CTX1, eluting between the two fractions, interacted differently with the column than CTX1B. It is necessary to keep in mind that C-CTX1 was naturally present in the sample and CTX1B was spiked; therefore, the column interaction of a toxin naturally incorporated into a matrix may not be the same as when the toxin was artificially added. However, this hypothesis was not evaluated in this research. Even so, this fact highlights the need to continue working to achieve other commercial CTX standards to make more appropriate analysis. On the other hand, it is still necessary (almost mandatory) to include these types of approximations to estimate the efficiency for CTX1B and CTX3C calculations, when studies using cleanup strategies and quantifications of CTXs are published.

**Figure 3:**
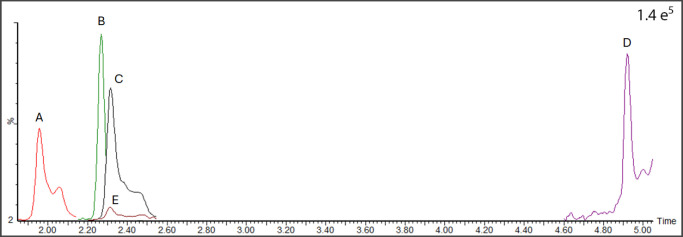
Extracted ion chromatograms (XICs) of the ciguatoxins (CTXs) involved in the present study and used as a reference for retention times, and ion ration confirmation, compared with a TIC of SPE fraction where C-CTX1 was detected. (A) 17-OH-C-CTX1 from a fish flesh (outbreak associated sample^41^ cleaned-up with second strategy (different Florisil batch)); (B) CTX1B solution standard from ILM; (C) C-CTX1 from a fish flesh (outbreak associated sample^41^ cleaned-up with second strategy (different Florisil batch)); (D) CTX3C solution standard from ILM; (E) sample example: F4 Flo-C18 SPE.

One aspect to highlight is the CTX3C assessment after SPEs. In general, the matrix effect for CTX3C was very high, when compared with CTX1B, except for the fractions considered as the filtrate or waste, which correspond, interestingly, to those resulting from the SPE in which Florisil cartridges were used, especially F1 from both Flo-C18 and Florisil strategies, with a matrix effect virtually nonexistent (ion suppression of 4% and 1%, respectively). Nevertheless, replication of these results may not be possible due to the variability in the performance of different lots of these cartridges.

Moreover, the quantifications of C-CTX1 in CTX1B equivalents, even in the extract before SPEs, when differences in matrix effects are not considered, allow an approximate evaluation of the recovery of the analyte after the different cleaning-up strategies when 2 μL were injected. Thus, it is important to denote that except for Flo-Amino SPE, all quantifications were quite similar ranging from 0.014 to 0.017 ng of CTX1B eq/g flesh (considering F1 and F2 from Florisil SPE together) (Table 1). When comparing the different toxicity recoveries in the fractions to their respective quantification of C-CTX1 by LC-MS/MS, only P.DBV-Silica and silica SPE toxicities stand out, suggesting that these last ones were able to recover nonmajor toxic analytes present in the raw flesh. Nevertheless, silica SPE would be a great cleanup approach for both analytical methods, CBA and LC-MS/MS, showing fractions with the highest toxicity and good recoveries for both CTX1B and CTX3C, although matrix enhancement of the CTX3C signal by LC-MS/MS may influence the quantification of other low-polar toxins such as CTX4A and CTX4B.

It is also worth mentioning that in fractions where C-CTX1 appeared, except for F1 Florisil and F2 Flo-Amino, a signal trace that matches with the retention time of 17-hydroxy-C-CTX1 was detected. However, their intensities were below the LOQ. This signal was taken into consideration since this analogue was first described in amberjack samples from the Canary Island waters,^41^ but no information about the toxicity of this compound is still available.

Even with the discrepancies in the efficiency of the different cleanup methods compared, all strategies were able to recover enough CTX analogues from a fish flesh sample near the EFSA/FDA suggested safety limit for consumption to be evidenced by CBA and LC-MS analysis. The results obtained by P.DVB-Silica and silica SPEs were highlighted as the most promising cleanup methods for the analysis of naturally contaminated samples. Besides, the finding of CTX-like toxicity in some filtered SPE fractions, usually treated as waste, indicates that the analysis of the different fractions by CBA is advantageous for evaluating the performance of the cleanup methods to be carried out.

By contrast, it seems that the cleanup strategies using Florisil cartridges are greatly influenced by the batch used. Therefore, it would be very helpful to use standard solutions of CTXs for calibration purposes and conduct analyses of their efficiency after changing the batch or brand. This could be very limiting for laboratories due to the high cost of CTX standards and reference materials.

Studies of recovery and matrix effect in CTXs are not economical, especially when they involve numerous analogues. Nonetheless, it is necessary to make the effort when analyzing toxins, such as CTXs, that cover a wide range of polarities in order to apply the recoveries to the quantifications with the highest accuracy possible.

As far as sample preparation is concerned, a chromatographic study of CTXs is tedious and time-consuming due to the need for implementing cleanup procedures. The effort increases if we consider the different fish samples to be treated, since not all extractions and purifications are equally efficient for the different matrices and the variety of possible analogues. This need limits the number of analyses a laboratory is capable of performing per week. On this basis, the flow of information and collaborations between different laboratories with expertise is necessary to achieve the selection of a method reaching consensus and being suitable for the analysis of the different CTXs.

## Supplementary Material



## Notes

Workflow followed to minimize variability due to individual extraction and spiking steps (Figure S1); in the upper part, chromatographic areas obtained for quantification transitions of CTX1B and CTX3C standards spiked in amberjack (Seriola sp.) blank matrix, both spiked CTXs eluted integrally in F2 of Florisil SPE; this trial was done as part of the earlier studies on the second cleanup strategy for assessment of the recovery and the matrix effect (with a different batch than the one used in the analysis described in this article); at the bottom, peaks of the C-CTXs (A: C-CTX1; B: 17-OH-C-CTX1) detected in MRM monitoring in different fish species matrix cleaned-up in previous tests carried out in different days for Florisil SPE (with a different Florisil batch) all of CTXs eluted in F2 of this strategy (Figure S2); multiple reaction monitored transitions for CTX1B in the different SPE strategies tested for matrix effect assessment (spiked at 10 ng CTX1B/mL) and standard solution: (A) CTX1B standard solution (10 ng CTX1B/mL), (B) F4 Florisil-C18 SPE, (C) F2 Florisil SPE, (D) F4 P.DVB-Silica SPE, (E) F1 Amino SPE, (F) F2 Silica SPE, and (G) F3 Flo-Amino SPE; the purple line corresponds to the CTX1B quantification signal (*m*/*z* 1128.6 > 95), the red line belongs to the pseudotransition of sodium (*m*/*z* 1133.6 > 1133.6), and the green line to the confirmation signal (*m*/*z* 1128.6 > 109); intensities were set at 1.50 e5 from B to G representations to facilitate visual comparison (Figure S3); multiple reaction monitored transitions for CTX3C in the different SPE strategies tested for matrix effect assessment (spiked at 10 ng CTX3C/mL), and standard solution: (A) CTX3C standard solution (14 ng CTX3C/mL), (B) F1 Florisil-C18 SPE, (C) F4 Florisil-C18 SPE, (D) F5 Florisil-C18 SPE, (E) F1 Florisil SPE, (F) F2 Florisil SPE, (G) F3 P.DVB-Silica SPE, (H) F4 P.DVB-Silica SPE, (I) Amino SPE, (J) F2 Silica SPE, (K) F2 Flo-amino, and (L) F3 Flo-Amino SPE; the purple line corresponds to the CTX3C quantification signal (*m*/*z* 1023.6 > 125.1), the red line belongs to the pseudotransition of sodium (*m*/*z* 1045.6 > 1045.6), and the green line corresponds to the confirmation signal (*m*/*z* 1023.6 > 155.1); all intensities are set to easily observe the sodium signal (*m*/*z* 1045.6 > 1045.6) of each sample (Figure S4); chromatographic conditions in Acquity UPLC I-Plus-Class (LC-MS/MS) (Table S1); multiple reaction monitoring method implemented in standards and reference material in the present study (Table S2); recovery (RE), matrix effect (ME), and efficiency for CTX1B and CTX3C in the different SPEs and quantification of C-CTX1 (expressed as ng eq CTX1B/mL) in the different fractions of each SPE (Table S3) (PDF)
