# Supplementary material for: Comparison of Different Solid-Phase Cleanup Methods Prior to the Detection of Ciguatoxins in Fish by Cell-Based Assay and LC-MS/MS
Source: J Agric Food Chem. 2025 Jun 2;73(23):14580–91. doi: 10.1021/acs.jafc.5c01142 (PMC12164330; doi:10.1021/acs.jafc.5c01142)
Supplement: Supplementary file 1 [file jf5c01142_si_001.pdf]

Suporting Information

Comparison of different solid phase clean-up methods prior to the detection of ciguatoxins in fish by cell-based assay and LC-MS/MS

Andres Sanchez-Henao[1,2*], Fernando Real[2], Yefermin Darias-Dágfeel[2], Natalia García-Álvarez[2], Jorge Diogène[1], Maria Rambla-Alegre[1*]

[1] IRTA, Marine and Continental Waters Program, Carretera de Poble Nou, 43540, La Ràpita, Catalonia, Spain

[2] IUSA, University Institute of Animal Health and Food Safety, University of Las Palmas de Gran Canaria, C/ Trasmontaña S/N, 35413, Arucas, Canarias Spain

**Correspondence: julianandres.sanchez@ulpgc.es and maria.rambla@irta.cat*

Figure S1. Workflow followed to minimize variability due to individual extraction and spiking steps.

Figure S2. In the upper part, chromatographic areas obtained for quantification transitions of CTX1B and CTX3C standards spiked in amberjack (*Seriola* sp.) blank matrix, both spiked CTXs eluted integrally in F2 of Florisil SPE. This trial was done as part of the earlier studies on the 2nd clean-up strategy for assessment of the recovery and the matrix effect (with a different batch than the one used in the analysis described in this article). At the bottom, peaks of the C-CTXs (**A**: C-CTX1; **B**: 17-OH-C-CTX1) detected in MRM monitoring in different fish species matrix cleaned-up in previous tests carried out in different days for Florisil SPE (with different Florisil batch) all CTXs eluted in F2 of this strategy.

Figure 32. Multiple Reaction Monitored transitions for CTX1B in the different SPE strategies tested for Matrix Effect assessment (spiked at 10 ng CTX1B/mL) and standard solution: (A) CTX1B standard solution (10 ng CTX1B/mL), (B) F4 Florisil-C18 SPE, (C) F2 Florisil SPE, (D) F4 P.DVB-Silica SPE, (E) F1 Amino SPE, (F) F2 Silica SPE, (G) F3 Flo-Amino SPE. Purple line corresponds to the CTX1B quantification signal (*m/z* 1128.6 > 95), red line belongs to the *pseudo-transition* of sodium (*m/z* 1133.6 > 1133.6), and green line to the confirmation signal (*m/z* 1128.6 > 109). Intensities were set at $1.50\ e^5$ from B to G representations to facilitate visual comparison.

Figure S4. Multiple Reaction Monitored transitions for CTX3C in the different SPE strategies tested for Matrix Effect assessment (spiked at 10 ng CTX3C/mL), and standard solution: (A) CTX3C standard solution (14 ng CTX3C/mL), (B) F1 Florisil-C18 SPE, (C) F4 Florisil-C18 SPE, (D) F5 Florisil-C18 SPE, (E) F1 Florisil SPE, (F) F2 Florisil SPE, (G) F3 P.DVB-Silica SPE, (H) F4 P.DVB-Silica SPE, (I) Amino SPE, (J) F2 Silica SPE, (K) F2 Flo-amino, and (L) F3 Flo-Amino SPE. Purple line corresponds to the CTX3C quantification signal (*m/z* 1023.6 > 125.1), red line belongs to the *pseudo-transition* of sodium (*m/z* 1045.6 > 1045.6), and green line corresponds to the confirmation signal (*m/z* 1023.6 > 155.1). All intensities are set to easily observe the sodium signal (*m/z* 1045.6 > 1045.6) of each sample.

Table S1. Chromatographic conditions in Acquity™ UPLC I-Plus-Class (LC-MS/MS).

Table S2. Multiple Reaction Monitoring method implemented in standards and reference material in the present study.

Table S3. Recovery (RE), Matrix Effect (ME) and Efficiency for CTX1B, CTX3C in the different SPEs; and quantification of C-CTX1 (expressed as ng Eq. CTX1B/mL) in the different fractions of each SPE.

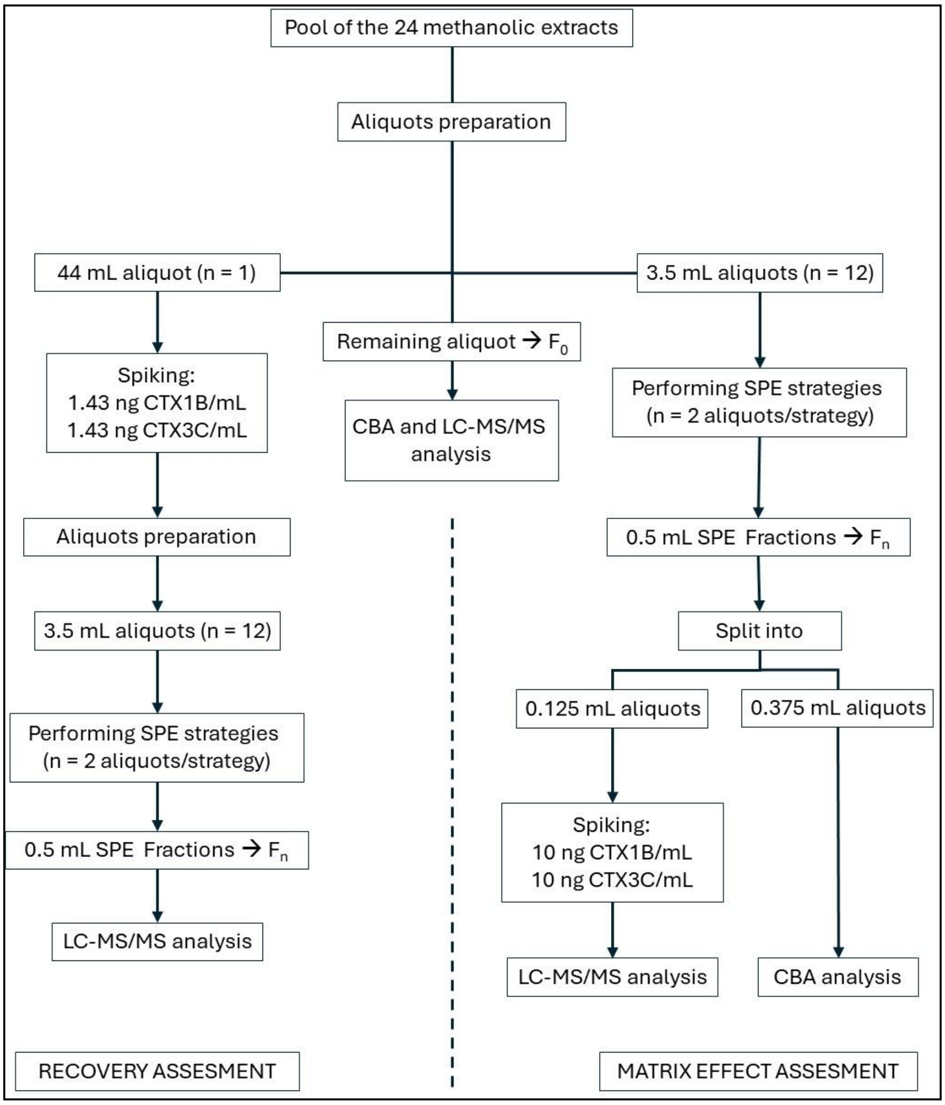

Figure S1. Workflow followed to minimize variability due to individual extraction and spiking steps.

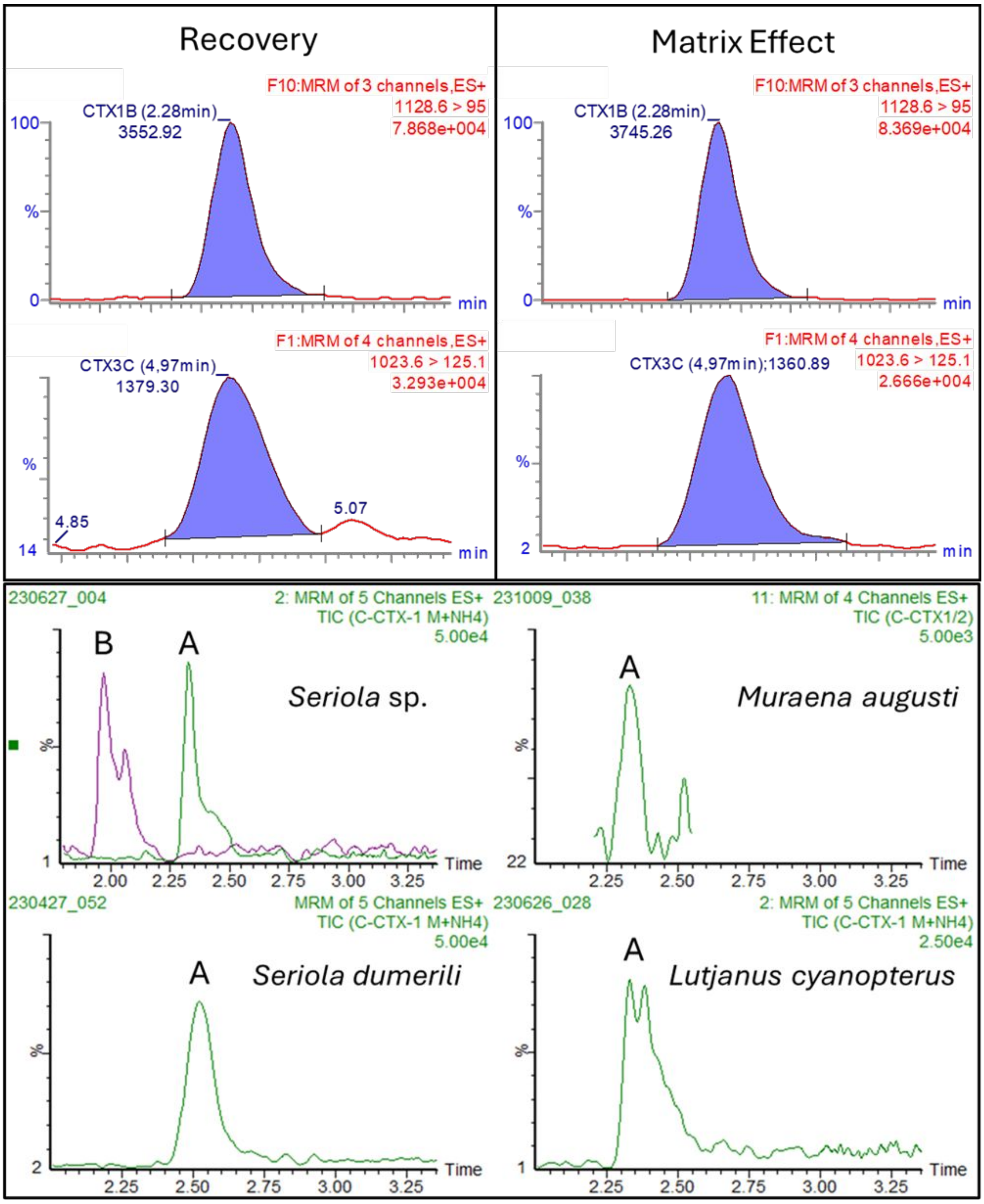

Figure S2. In the upper part, chromatographic areas obtained for quantification transitions of CTX1B and CTX3C standards spiked in amberjack (*Seriola* sp.) blank matrix, both spiked CTXs eluted integrally in F2 of Florisil SPE. This trial was done as part of the earlier studies on the 2nd clean-up strategy for assessment of the recovery and the matrix effect (with a different batch than the one used in the analysis described in this article). At the bottom, peaks of the C-CTXs (**A**: C-CTX1; **B**: 17-OH-C-CTX1) detected in MRM monitoring in different fish species matrix cleaned-up in previous tests carried out in different days for Florisil SPE (with different Florisil batch) all CTXs eluted in F2 of this strategy.

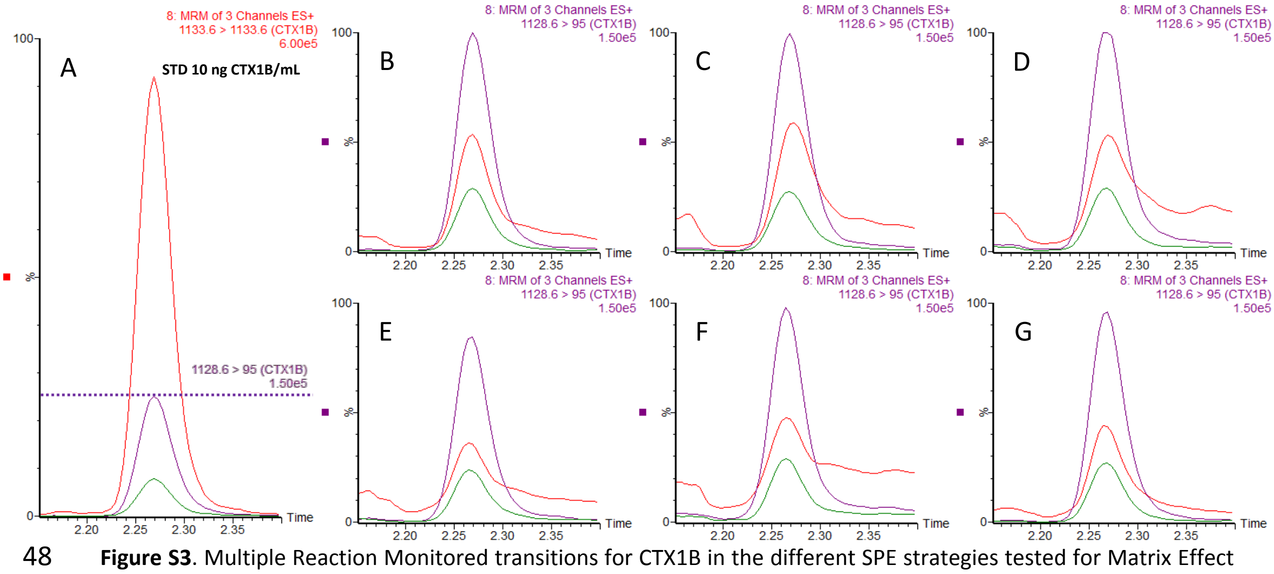

Figure S3. Multiple Reaction Monitored transitions for CTX1B in the different SPE strategies tested for Matrix Effect assessment (spiked at 10 ng CTX1B/mL) and standard solution: (**A**) CTX1B standard solution (10 ng CTX1B/mL), (**B**) F4 Florisil-C18 SPE, (**C**) F2 Florisil SPE, (**D**) F4 P.DVB-Silica SPE, (**E**) F1 Amino SPE, (**F**) F2 Silica SPE, (**G**) F3 Flo-Amino SPE. Purple line corresponds to the CTX1B quantification signal (*m/z* 1128.6 > 95), red line belongs to the *pseudo-transition* of sodium (*m/z* 1133.6 > 1133.6), and green line to the confirmation signal (*m/z* 1128.6 > 109). Intensities were set at 1.50 e^5 from B to G representations to facilitate visual comparison.

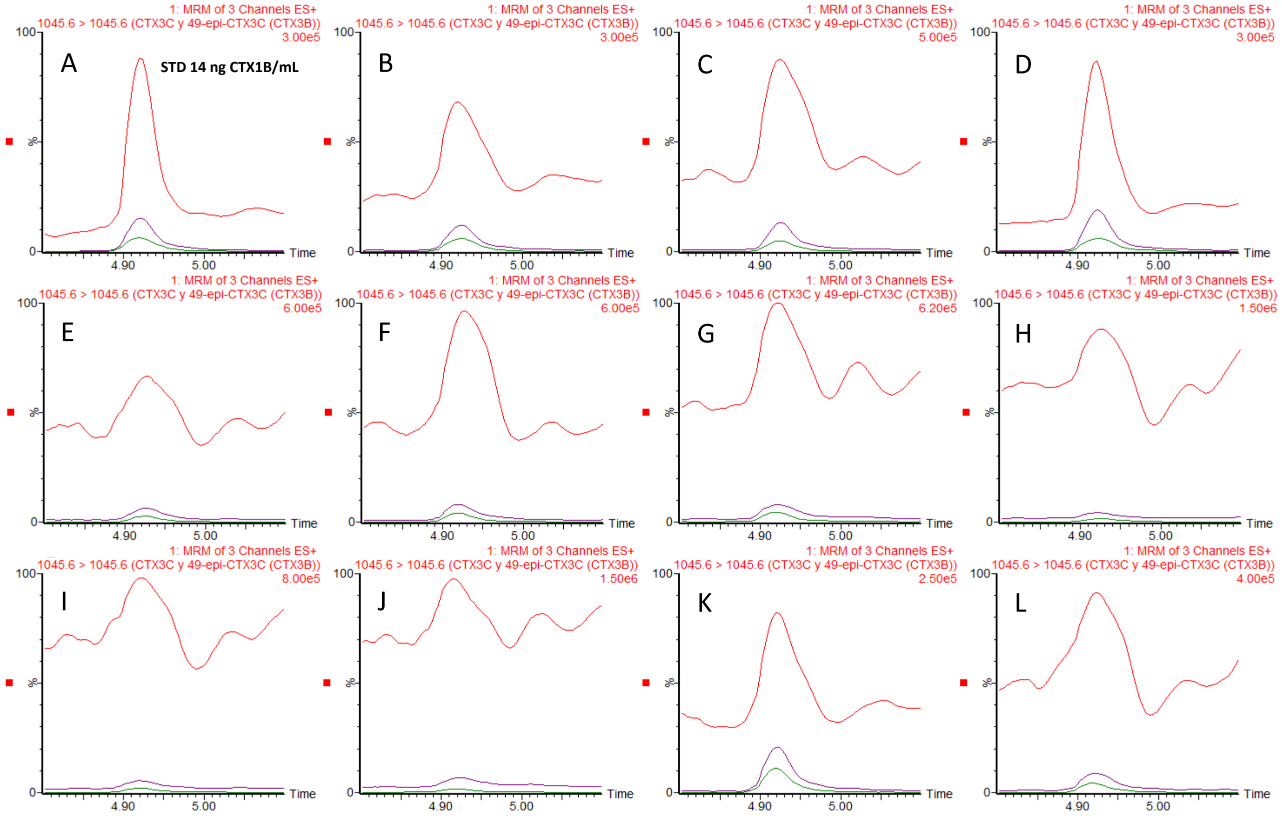

Figure S4. Multiple Reaction Monitored transitions for CTX3C in the different SPE strategies tested for Matrix Effect assessment (spiked at 10 ng CTX3C/mL), and standard solution: (**A**) CTX3C standard solution (14 ng CTX3C/mL), (**B**) F1 Florisil-C18 SPE, (**C**) F4 Florisil-C18 SPE, (**D**) F5 Florisil-C18 SPE, (**E**) F1 Florisil SPE, (**F**) F2 Florisil SPE, (**G**) F3 P.DVB-Silica SPE, (**H**) F4 P.DVB-Silica SPE, (**I**) Amino SPE, (**J**) F2 Silica SPE, (**K**) F2 Flo-amino, and (**L**) F3 Flo-Amino SPE. Purple line corresponds to the CTX3C quantification signal (*m/z* 1023.6 > 125.1), red line belongs to the *pseudo-transition* of sodium (*m/z* 1045.6 > 1045.6), and green line corresponds to the confirmation signal (*m/z* 1023.6 > 155.1). All intensities are set to easily observe the sodium signal (*m/z* 1045.6 > 1045.6) of each sample.

Table S1. Chromatographic conditions in Acquity™ UPLC I-Plus-Class (LC-MS/MS).

**Time** (min)	**Mobile phase A** H2O + 0.2 mM of ammonium formate + 0.1% formic acid	**Mobile phase B** Acetonitrile- H2O (95:5) + 0.2 mM of ammonium formate + 0.1% formic acid	**Flow** (mL/min)
0	95%	5%	0.4
1	50%	50%	0.4
5	0%	100%	0.4
7	0%	100%	0.4
7.01	95%	5%	0.4
11	95%	5%	0.4

Table S2. Multiple Reaction Monitoring method implemented in standards and reference material in the present study.

CTX analogue	Transition	Parent (*m/z*)	Daughter (*m/z*)	Cone (V)	Collision (eV)
CTX1B	$[M+NH_4]^+$ > 95*	1128.6	95	20	80
	$[M+NH_4]^+$ > 109	1128.6	109	20	80
	$[M+Na]^+$ > $[M+Na]^+$	1133.6	1133.6	20	20
CTX2 and CTX3	$[M+NH_4]^+$ > $[M+H-H_2O]^+$*	1112.6	1077.6	20	20
	$[M+NH_4]^+$ > $[M+H-2H_2O]^+$	1112.6	1059.6	20	20
CTX4A and CTX4B	$[M+H]^+$ > 125.1*	1061.6	125.1	20	70
	$[M+NH_4]^+$ > $[M+H-H_2O]^+$	1078.6	1043.6	20	15
CTX3C and CTX3B	$[M+H]^+$ > 125.1*	1023.6	125.1	20	70
	$[M+H]^+$ > 155.1	1023.6	155.1	20	70
	$[M+Na]^+$ > $[M+Na]^+$	1045.6	1045.6	20	20
M-seco-CTX3C	$[M+H]^+$ > $[M+H-H_2O]$*	1041.6	1023.6	20	20
	$[M+H]^+$ > $[M+H-2H_2O]$	1041.6	1005.6	20	20
2,3-dyhidroxy-CTX3C	$[M+NH_4]^+$ > $[M+H-H_2O]^+$*	1074.6	1039.6	20	20
	$[M+NH_4]^+$ > $[M+H-2H_2O]^+$	1074.6	1021.6	20	30
51-hydroxy-CTX3C	$[M+NH_4]^+$ > $[M+H-H_2O]^+$*	1056.6	1021.6	20	20
	$[M+NH_4]^+$ > $[M+H-2H_2O]^+$	1056.3	1003.6	20	20
C-CTX1	$[M+NH_4]^+$ > $[M+H-H_2O]^+$*	1158.6	1123.6	20	20
	$[M+NH_4]^+$ > $[M+H-H_2O]^+$	1158.6	1005.6	20	20
	$[M+NH_4]^+$ > 109.1	1158.6	109.1	20	70
	$[M+NH_4]^+$ > 191.1	1158.6	191.1	20	70
17-hydroxy-C-CTX1	$[M+NH_4]^+$ > $[M+H-H_2O]^+$*	1174.6	1139.6	20	20
	$[M+NH_4]^+$ > 191.1	1174.6	191.1	20	70
	$[M+H-H_2O]^+$ > $[M+H-2H_2O]^+$	1139.6	1121.6	20	20
	$[M+H-H_2O]^+$ > 191.1	1139.6	109.1	20	70
C-CTX3 and C-CTX4	$[M+H]^+$ > $[M+H-H_2O]$*	1143.6	1125.6	20	20
	$[M+H]^+$ > 108.9	1143.6	108.9	20	70

(*) Quantification transition signal.

Table S3. Recovery (RE), Matrix Effect (ME) and Efficiency for CTX1B, CTX3C in the different SPEs; and quantification of C-CTX1 (expressed as ng Eq. CTX1B/mL) in the different fractions of each SPE.

SPE stragetgy	Fraction (F_n)	**CTX1B**				**CTX3C**				**C-CTX1**[1]	
		CTX1B (ng/mL)	SD (ng/mL)	Recovery (RE) or matrix effect (ME)	SPE efficiency (%)	CTX3C (ng/mL)	SD (ng/mL)	Recovery (RE) or matrix effect (ME)	SPE efficiency (%)	C-CTX1 (ng Eq CTX1B/ mL)	SD (ng Eq CTX1B/ mL)
1st Florisil-C18	1	ND	-	RE: 0%	0%	7.73	0.41	RE: 77%	81%	ND	-
		8.54	0.09	ME: 85%		9.61	1.68	ME: 96%			
	4	7.44	0.22	RE: 74%	72%	3.36	0.31	RE: 34% *	20% *	0.27	0.05
		10.28	0.09	ME: 103%		17.17	0.98	ME: 172%			
	5	ND	-	RE: 0%	0%	2.04	0.32	RE: 20%	14%	ND	-
		10.99	0.45	ME: 110%		14.84	0.75	ME: 148%			
2nd Florisil	1	ND	-	RE: 0%	0%	10.15	0.56	RE: 101%	103%	0.08[2]	0.02
		8.59	0.26	ME: 86%		9.89	0.43	ME: 99%			
	2	6.52	0.22	RE: 65%	67%	ND	-	RE: 0%	0%	0.22	0.04
		9.77	0.21	ME: 98%		13.02	0.49	ME: 130%			
3rd P.DVB-Silica	3	ND	-	RE: 0%	0%	ND	-	RE: 0%	0%	ND	-
		11.16	0.68	ME:112%		16.35	1.96	ME: 163%			
	4	7.80	0.39	RE: 78%	79%	11.14	0.81	RE: 111%	85%	0.29	0.06
		9.89	0.36	ME: 99%		13.10	1.06	ME: 131%			
4th Amino	1	7.91	0.25	RE: 79%	93%	9.66	0.36	RE: 97%	86%	0.26	0.03
		8.47	0.17	ME: 85%		11.29	1.34	ME: 113%			
5th Silica	2	7.61	0.14	RE: 76%	86%	17.03	0.82	RE: 164%	90%	0.24	0.06
		8.89	0.13	ME: 89%		18.29	0.79	ME: 186%			
6th Flo-Amino	2	ND	-	RE: 0%	0%	7.10	1.15	RE: 71%	51%	ND	-
		10.05	0.22	ME:101%		13.79	0.97	ME: 138%			
	3	3.14	0.21	RE: 31%	34%	3.96	0.97	RE: 40%	35%	0.12	0.02
		9.15	0.15	ME: 92%		11.22	2.25	ME: 112%			

(*) These recovery and efficiency values were ruled out due to the ion ratio criteria, (difference > 35%) compared to STD values. (SD) Standard deviation. ([1]) as no commercial standard is available, only data of chromatographic areas in ng Eq. of CTX1B/mL are indicated. ([2]) Quantification off calibration curve.

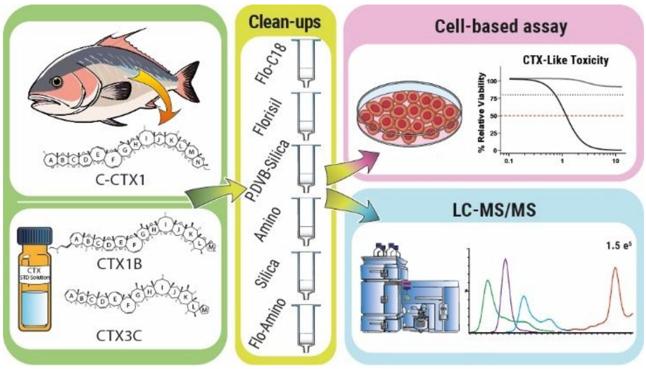
C-CTX1
CTX1B
CTX3C
Clean-ups
Flo-C18
Florisil
PDVB-Silica
Amino
Silica
Flo-Amino
Cell-based assay
CTX-Like Toxicity
% Relative Viability
LC-MS/MS
1.5 e5
